# Impact of predictive medicine on therapeutic decision making: a randomized controlled trial in congenital heart disease

**DOI:** 10.1038/s41746-019-0085-1

**Published:** 2019-03-19

**Authors:** Huseyin Naci, Maximilian Salcher-Konrad, Alistair Mcguire, Felix Berger, Titus Kuehne, Leonid Goubergrits, Vivek Muthurangu, Ben Wilson, Marcus Kelm

**Affiliations:** 1 LSE Health, Department of Health Policy, London School of Economics and Political Science, London, UK; 2 German Heart Institute Berlin (DHZB), Berlin, Germany; 3 Charité – Universitätsmedizin Berlin, Pediatric Cardiology, Berlin, Germany; 4 DZHK (German Centre for Cardiovascular Research), partner site Berlin, Berlin, Germany; 5 Institute for Computational and Imaging Science in Cardiovascular Medicine, Charité – Universitätsmedizin Berlin, corporate member of Freie Universität Berlin, Humboldt-Universität zu Berlin, and Berlin Institute of Health, Berlin, Germany; 6 Great Ormond Street Hospital, University College London, London, UK; 7 Department of Sociology, Stockholm University, Stockholm, Sweden; 8 Department of Methodology, London School of Economics and Political Science, London, UK

**Keywords:** Health policy, Congenital heart defects

## Abstract

Computational modelling has made significant progress towards clinical application in recent years. In addition to providing detailed diagnostic data, these methods have the potential to simulate patient-specific interventions and to predict their outcome. Our objective was to evaluate to which extent patient-specific modelling influences treatment decisions in coarctation of the aorta (CoA), a common congenital heart disease. We selected three cases with CoA, two of which had borderline indications for intervention according to current clinical guidelines. The third case was not indicated for intervention according to guidelines. For each case, we generated two separate datasets. First dataset included conventional diagnostic parameters (echocardiography and magnetic resonance imaging). In the second, we added modelled parameters (pressure fields). For the two cases with borderline indications for intervention, the second dataset also included pressure fields after virtual stenting simulations. All parameters were computed by modelling methods that were previously validated. In an online-administered, invitation-only survey, we randomized 178 paediatric cardiologists to view either conventional (control) or add-on modelling (experimental) datasets. Primary endpoint was the proportion of participants recommending different therapeutic options: (1) surgery or catheter lab (collectively, “intervention”) or (2) no intervention (follow-up with or without medication). Availability of data from computational predictive modelling influenced therapeutic decision making in two of three cases. There was a statistically significant association between group assignment and the recommendation of an intervention for one borderline case and one non-borderline case: 94.3% vs. 72.2% (RR: 1.31, 95% CI: 1.14–1.50, *p* = 0.00) and 18.8% vs. 5.1% (RR: 3.09, 95% CI: 1.17–8.18, *p* = 0.01) of participants in the experimental and control groups respectively recommended an intervention. For the remaining case, there was no difference between the experimental and control group and the majority of participants recommended intervention. In sub-group analyses, findings were not affected by the experience level of participating cardiologists. Despite existing clinical guidelines, the therapy recommendations of the participating physicians were heterogeneous. Validated patient-specific computational modelling has the potential to influence treatment decisions. Future studies in broader areas are needed to evaluate whether differences in decisions result in improved outcomes (Trial Registration: NCT02700737).

## Introduction

Health systems are increasingly aiming to adopt precision medicine to better account for the well-characterized heterogeneity among patient populations.^1^ In line with these goals, diagnostic methods, such as imaging, have improved substantially over the past years and are able to characterize patients at an ever increasing granularity. At the same time, recommendations in clinical practice guidelines led to major advances in evidence-based medicine.^2^ However, such guidelines are often inadequate to facilitate patient-centred care as they primarily aim to standardize treatment and therefore account only partially for inter-individual variability.^3^ This often leaves clinicians with significant uncertainty when choosing the optimal treatment strategy.

In coarctation of the aorta (CoA), a relatively common congenital heart disease, decisions on the timing and type of treatment (wait vs. pharmacological treatment vs. intervention) are crucial to prevent long-term sequalae such as persistent arterial hypertension and end-organ damage.^4^ However, recommendations do not always agree across different guidelines and thus leave room for debate, especially in clinically borderline cases. For example, the American College of Cardiology and the American Heart Association recommend intervention for primary CoA or restenosis if invasive peak-to-peak pressure gradients across the CoA exceed 20 mmHg with or without significant narrowing.^5^ On the other hand, the European Society of Cardiology recommends interventional treatment if pressure gradients are higher than 20 mmHg based on cuff pressures between upper and lower limbs in the presence of arterial hypertension.^6^

Patient-specific modelling has the potential to support individual therapy decisions and improve the success rate of interventions. For the evaluation of CoA, computational fluid dynamic (CFD) methods are of particular interest as they provide valuable information about hemodynamics such as pressure fields and flow profiles.^7,8^ In addition, they can be coupled with virtual intervention tools such as stenting of the CoA or aortic valve replacement which allow to predict the immediate hemodynamic effects of a given intervention.^9,10^ Therefore, such models can provide diagnostic as well as prognostic information concerning the hemodynamics before or after the simulated intervention. In turn, availability of patient-specific modelling data can reduce two key sources of uncertainty for clinical decision-making—diagnosis and prognosis—potentially influencing treatment recommendations.^11^

Despite recent advances of such patient-specific computational modelling concepts, the potential for clinical translation has not been subject to extensive evaluation. In recent years, there have been repeated calls to evaluate the effectiveness and cost-effectiveness of these approaches in larger populations.^12,13^ However, an important first step is to evaluate their potential impact on clinician behaviour and decision making. Without demonstrable changes in clinicians’ treatment decisions as a result of improved information garnered through computational modelling, subsequent changes in patient outcomes are unlikely.

Our objective was to design and implement a randomized controlled trial to evaluate whether and to what extent patient-specific computational modelling influences clinical treatment decisions in a common congenital heart condition (CoA). To do so, we designed an experiment with different levels of data presented to cardiologists to assess whether viewing image-based, patient-specific modelling altered the recommended course of action in hypothetical treatment scenarios.

## Results

### Participant characteristics

**Fig. 1:**
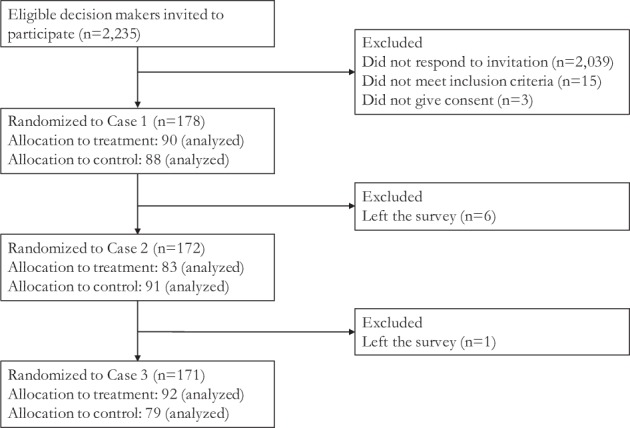
Flow of participants in the trial

Figure 1 shows the flow of participants in the study. Of 2235 eligible clinicians invited to participate, 2039 did not respond; 15 did not meet the inclusion criteria (either participated in CARDIOPROOF or had no experience treating patients with coarctation of the aorta over the past 6 months); and three did not give consent. A total of 178 cardiologists participated; 90 were randomly allocated to receive patient-specific computational modelling data for the first case (experimental) and 88 received conventional imaging parameters (control). After completing the questionnaire for the first case, 6 participants left the study, leaving 172 participants for the second case. Another participant left the survey after completing the questionnaire for the second case. A total of 171 participants were randomized to the third case: 92 and 79 participants were randomized to experimental and control groups, respectively.

Participants in experimental and control arms of the trial had similar baseline characteristics. For the first two cases, there were no detectable differences between the groups (Table 1). There were only a few statistically significant between-group differences for the third case. For example, 87.3% of participants in the control group had over five years of experience with decision making in congenital heart disease as compared to 71.4% of those in the experimental group (*p* = 0.01). Similarly, 88.8% of the participants in the control group had treatment experience on more than five coarctation of the aorta cases in the past year as compared to 76.7% of those in the experimental group (*p* = 0.04).

**Table 1:** Baseline characteristics of trial participants

	Experimental group: Conventional & modelling data	Control group: Conventional data	*p*-value for difference
*Case 1*	*n* = 90	*n* = 88	
Gender	34.5	35.8	0.86
Proportion female (95% CI)	(24.3–44.7)	(25.1–46.5)	
Region	69.0	69.1	0.98
Proportion practicing in Western Europe (95% CI)	(59.0–78.9)	(58.9–79.4)	
CHD experience	81.8	75.3	0.29
Proportion with >5 years of experience with decision making in CHD (95% CI)	(73.6–90.0)	(66.1–84.4)	
CoA experience	85.2	80.7	0.43
Proportion with treatment experience on >5 CoA cases in the past year (95% CI)	(77.7–92.8)	(72.3–89.1)	
Catheter intervention experience	38.6	29.5	0.21
Proportion with experience in performing catheter interventions on >5 cases per year (95% CI)	(28.3–49.0)	(19.8–39.3)	
*Case 2*	*n* = 83	*n* = 89	
Gender	36.3	34.1	0.77
Proportion female (95% CI)	(25.5–47.0)	(23.9–44.2)	
Region	65.0	72.7	0.28
Proportion practicing in Western Europe (95% CI)	(54.3–75.7)	(63.2–82.2)	
CHD experience	80.7	77.3	0.58
Proportion with >5 years of experience with decision making in CHD (95% CI)	(72.1–89.4)	(68.3–86.2)	
CoA experience	78.3	86.2	0.18
Proportion with treatment experience on >5 CoA cases in past year (95% CI)	(69.3–87.4)	(78.8–93.6)	
Catheter intervention experience	34.9	34.5	0.95
Proportion with experience in performing catheter interventions on >5 cases per year (95% CI)	(24.5–45.4)	(24.3–44.7)	
*Case 3*	*n* = 92	*n* = 79	
Gender	35.6	34.6	0.89
Proportion female (95% CI)	(25.5–45.6)	(23.8–45.4)	
Region	71.1	66.7	0.54
Proportion practicing in Western Europe (95% CI)	(61.6–80.7)	(56.0–77.4)	
CHD experience	71.4	87.3	0.01*
Proportion with >5 years of experience with decision making in CHD (95% CI)	(62.0–80.9)	(79.8–94.8)	
CoA experience	76.7	88.8	0.04*
Proportion with treatment experience on >5 CoA cases in the past year (95% CI)	(67.8–85.6)	(81.4–95.8)	
Catheter intervention experience	36.7	32.9	0.62
Proportion with experience in performing catheter interventions on >5 cases per year (95% CI)	(26.5–46.8)	(22.3–43.5)	

*CHD* congenital heart disease, *CoA* coarctation of the aorta, *CI* confidence interval

*Statistically significant at 0.05 level

### Outcomes

**Fig. 2:**
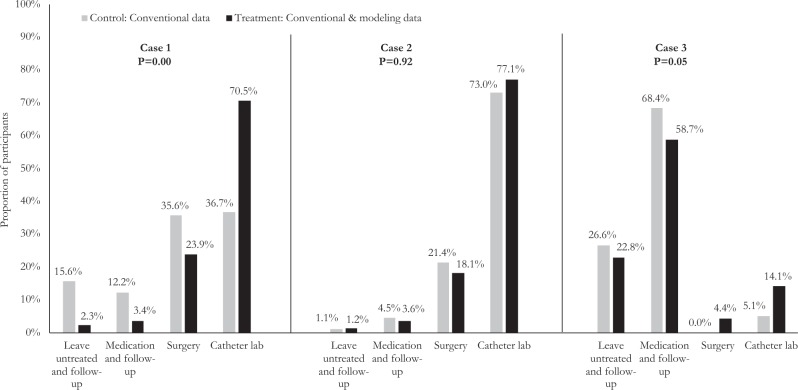
Recommended course of action for each case. Experimental group includes participants randomized to see patient-specific modelling results in addition to conventional imaging data. Control group includes participants randomized to see only conventional imaging data. Fisher’s exact test was used to statistically test for an association between group assignment and recommended course of action. *p*-value for case 1: 0.00; case 2: 0.92; and case 3: 0.05. *p*-value < 0.05 indicates a statistically significant difference between the groups

Figure 2 shows the recommended course of action for each case. For the first case, overall, there was a statistically significant association between group assignment and recommended course of action (*p* = 0.00). For example, 70.5% of participants who were presented with patient-specific modelling data in addition to conventional parameters recommended referring the patient to the catheter lab as compared to 36.7% of participants who were given conventional parameters alone. Fewer participants in the experimental group recommended surgery compared to those in the control group (23.9% vs. 35.6%, respectively). In addition, fewer participants in the experimental group recommended no intervention (leave untreated and follow-up, 2.3%; follow-up with medication, 2.4%) compared to those in the control group (leave untreated and follow-up, 15.6%; follow-up with medication, 12.2%). For the second case, there was no discernible difference between the groups in terms of their recommended course of action (*p* = 0.92). A similar proportion of participants recommended referring the patient to the catheter lab (77.1% in treatment vs. 73.0% in control). A marginally statistically significant difference was observed between the two groups for the third case (*p* = 0.05).

Findings of the primary analysis are shown in Table 2. A higher proportion of participants recommended an intervention (either surgery or catheter lab) when presented with patient-specific modelling data in addition to conventional parameters for the first case (borderline) and third case (non-borderline). For the first case, 94.3% of participants in the experimental group recommended an intervention as opposed to 72.2% of those in the control group (relative risk, RR: 1.31, 95% CI: 1.14, 1.50, *p* = 0.00); for the third case, 18.5% of participants in the experimental group and 5.1% of participants in the control group recommended an intervention (RR: 3.09, 95% CI: 1.17, 8.18, *p* = 0.01). There was no statistically significant difference between the participants who were and were not presented with patient-specific computational modelling data for the second case; 95.2% vs. 94.4% of those in the experimental group recommended an intervention (RR: 1.00, 95% CI: 0.94, 1.07, *p* = 0.82).

**Table 2:** Proportion of participants recommending an intervention

	No. recommending intervention/total (%)		
	Experimental group (Conventional & modelling data)	Control group (Conventional data)	Relative risk (95% CI)
Case 1	83/88 (94.3)	65/90 (72.2)	1.31 (1.14, 1.50)
Case 2	79/83 (95.2)	84/89 (94.4)	1.00 (0.94, 1.07)
Case 3	17/92 (18.5)	4/79 (5.1)	3.09 (1.17, 8.18)

*CI* confidence interval

Differences in the proportions of participants recommending either surgical intervention or catheter lab as opposed to no intervention (follow-up with or without medication) in experimental vs. control groups did not differ according to experience level (Table 3). For example, more participants recommended an intervention for the first case when they were presented with patient-specific modelling data in addition to conventional parameters compared to participants presented with only conventional imaging data, regardless of whether they had more than 5 years of experience in congenital heart disease (RR: 1.27, 95% CI: 1.09, 1.49) or less than 5 years of experience (RR: 1.47, 95% CI: 1.10, 1.95).

**Table 3:** Proportion of participants recommending an intervention according to experience level

	No. recommending intervention/Total (%)		
	Experimental group (Conventional & modelling data)	Control group (Conventional data)	Relative risk (95% CI)
*Case 1*			
More CHD experience	67/72 (93.1)	49/67 (73.1)	1.27 (1.09, 1.49)
Less CHD experience	16/16 (100.0)	15/22 (68.2)	1.47 (1.10, 1.95)
More CoA experience	72/75 (96.0)	52/71 (73.2)	1.31 (1.13, 1.52)
Less CoA experience	11/13 (84.6)	11/17 (64.7)	1.31 (0.86, 1.99)
More catheter intervention experience	32/34 (94.1)	21/26 (80.8)	1.16 (0.96, 1.43)
Less catheter intervention experience	51/54 (94.4)	42/62 (67.7)	1.39 (1.16, 1.68)
*Case 2*			
More CHD experience	63/67 (94.0)	64/68 (94.1)	0.99 (0.92, 1.09)
Less CHD experience	16/16 (100.0)	19/20 (95.0)	1.05 (0.95, 1.16)
More CoA experience	62/65 (95.4)	71/75 (94.7)	1.01 (0.93, 1.09)
Less CoA experience	17/18 (94.4)	11/12 (91.7)	1.03 (0.84, 1.26)
More catheter intervention experience	28/29 (96.6)	29/30 (96.7)	0.99 (0.91, 1.09)
Less catheter intervention experience	51/54 (94.4)	53/57 (93.0)	1.02 (0.92, 1.12)
*Case 3*			
More CHD experience	9/65 (13.8)	2/69 (2.9)	4.75 (1.07, 21.29)
Less CHD experience	8/26 (30.8)	2/10 (20.0)	1.54 (0.39, 6.04)
More CoA experience	13/69 (18.8)	4/70 (5.7)	3.29 (1.13, 9.61)
Less CoA experience	4/21 (19.0)	0/9 (0.0)	Not estimable
More catheter intervention experience	6/33 (18.2)	1/26 (3.8)	4.79 (0.60, 36.86)
Less catheter intervention experience	11/57 (19.3)	3/53 (5.7)	3.39 (1.01, 11.56)

More experience refers to (1) >5 years of experience with decision making in congenital heart disease; (2) >5 coarctation of the aorta cases in past year; and (3) >5 catheter interventions per year

*CHD* congenital heart disease, *CoA* coarctation of the aorta, *CI* confidence interval

## Discussion

In this randomized controlled trial, we evaluated the treatment recommendations of physicians that were either based on current state-of-the-art diagnostic information or additional information from patient-specific computational models. We observed that despite existing clinical guidelines, the therapy recommendations of the participating physicians were quite heterogeneous for the individual patients when based on “conventional” diagnostic information. Patient-specific models appear to influence the recommended course of action. Cardiologists who were presented with computational modelling data in addition to conventional echocardiography and MRI data were more likely to recommend surgery or catheter intervention for two cases (one borderline and non-borderline). These findings were not affected by the experience level of the participating cardiologists.

Research on the development of integrated computer models of the mechanical, physical and biochemical functions of a living human body has drawn significant attention and has driven investment for the past decade.^14–16^ This investment has been warranted on the premise that individualized risk prediction and virtual treatment planning have the potential to improve patient outcomes in many diseases.^17^ Indeed, previous models raised significant expectations to make decision support tools available for early diagnosis, disease prediction and outcome optimization. Recent studies increasingly provide supporting evidence on the clinical reliability and validity of models emanating from these research efforts.^18–21^

The applied models in our study were validated in previous work.^7,8,10^ Uncertainties were addressed and it was shown that they can provide diagnostic information about pressure maps that are equivalent to invasive cardiac catheterization.^22,23^ In addition, it was shown that haemodynamic models after virtual treatment procedures can predict the hemodynamic effects of intervention.^10^ In the present study, for both cases that received stent placement there was good agreement between predicted pressure drop and pressure drop measured by catheter post-intervention. However, investigated modelling methods are not meant to replace evidence-based guidelines that are based upon mid- or long-term outcome data. Instead, the modelling methods allow simulating the immediate hemodynamic effects of an intervention and thus may help answering whether a given intervention will provide hemodynamically meaningful results. This a-priori knowledge can be of clinical value, but such novel methods must be introduced into the clinical setting cautiously. It not only requires methods to be validated, but also to obtain knowledge about the impact of computational models on clinical decision-making process. This is what our trial starts to address.

Currently, little is known about how clinicians comprehend and utilize new patient-specific data, and whether this influences their treatment decisions.^24–26^ In this trial, we show that physicians who have access to patient-specific modeling data make different treatment recommendations. These findings are important to establish the decision impact of modelling data before investing in sizeable outcome trials in large patient populations. Future large trials should evaluate the appropriateness of making different treatment recommendations by mapping treatment decisions to mid-term and to long-term clinical outcomes. The design and findings of our scenario-based randomized experiment can offer insights for future evaluations of computational modelling approaches.

In accordance with what is widely known in clinical practice, we noted significant heterogeneity in decision making within each of the clinical cases. This result highlights the limitations of clinical guidelines in this area, despite their indisputable role in evidence-based medicine. In contrast, the use of the patient-specific models can result in distinctive shifts in decisions. This underscores the promises as well as the risks of computational models for personalized medicine. One can assume that it will be difficult for a user to deviate from the recommendation of a model-based decision support system if it predicts a given effect of an intervention. Advocates of patient-specific computational modelling often argue that individualizing treatment strategies would optimize the type and timing of interventions, which in turn reduce unnecessary or inappropriate invasive procedures, and help realize substantial cost savings.^27,28^

How did patient-specific modelling data influence treatment recommendations? We had anticipated that patient-specific modelling data would reduce uncertainty around diagnosis and prognosis, thereby resulting in different treatment recommendations. In our trial, we presented computational modelling data on both the diagnosis and prognosis for two cases (Cases 1 and 2), and only on the diagnosis for one case (Case 3). Statistically significantly different recommendations were observed for Cases 1 and 3, indicating that it was reduced uncertainty—irrespective of its source—that was responsible for influencing treatment recommendations. In addition to the effect of reduced uncertainty, clinical decision making processes could be influenced by several other factors. These factors include participants’ experience, hospital-specific practice, framing, search satisficing or even very subtle factors such as the scaling of the axes of graphs that have been extensively studied previously.^29–31^

In our study we have presented the participants primarily CFD-based models since hemodynamic parameters such as the distribution of pressure gradients are particularly important for CoA. The CFD models have also the advantage that the results are relatively easy to visualize for the user which is important for the straightforward implementation of the trial. In addition, the CFD models can also be used together with tools that allow performing virtual interventions.^9^ In the current study, we focused on virtual stent implantation to keep the experimental setup simple. The representation of surgical interventions is technically also possible,^10^ but would have increased the complexity of the trial set-up. In contrast, models that allow to assess the patient-specific response to pharmacological treatment are still under development and thus were also not used in our trial.

Providing the participants of our study with simulations obtained after virtual stenting for the first two cases may have primed them to consider the potential effects of stenting as opposed to other treatment alternatives. However, the proportion of participants who recommended referring patients to the catheter lab was not higher for Case 2 despite the availability of virtual stenting information. In addition, more participants recommended intervention for Case 3 despite no virtual stenting data presented for this case. These suggest that the priming effect of presenting virtual stenting data may be modest. Moreover, we cannot exclude that the presentation style of the pre-interventional hemodynamic results could have influenced the participants. For Case 3, the graph of the pre-interventional hemodynamics shows a sharp pressure drop across the stenosis. In addition, the *x*-axes had a limited range which could have implied a relatively large pressure drop. These could be reasons why participants with model-based information (compared to participants provided only with conventional information) recommended an intervention in this case, despite the fact that the patient had no formal indication for intervention according to existing clinical guidelines. However, these explanations remain speculative. Therefore, future studies should be conducted that assess systematically how specific factors of modelling (including the presentation of modelling results) can shift decision in a given direction.

The findings of our study should be interpreted in light of its limitations. We used data from three cases. Although a small number, these cases were carefully selected by experienced clinicians to demonstrate heterogeneity in clinical presentation and indication for intervention. Second, clearly this is an experimental study and we only considered hypothetical treatment decisions. It is conceivable that our study participants deviated from the types of decisions that they would have made in actual clinical settings. However, the extent to which their responses were affected by the hypothetical nature of our experiment should be similar in both the treatment and control groups, and therefore should not influence our findings. In addition, generalizability of our findings should be investigated further. Of 2235 cardiologists invited to take part in this study, 2039 did not respond. Another key limitation of our study is its sample. Cardiologists who agreed to participate represent a selected sample of potential users of patient-specific modelling approaches and may have a higher degree of knowledge and curiosity about modeling approaches. It is possible, therefore, participants of this trial had different attitudes towards innovative technologies (and hence treatment recommendations) than those who declined to take part.

In conclusion, our randomized experiment provides insights into the potential feasibility and clinical utility of novel modelling approaches aimed at individualizing treatment decisions for congenital heart conditions. This evaluation, despite focusing on a small number of cases, is nonetheless supportive of the use of individual patient computational modeling in arriving at a clinical decision. Whether observed differences in treatment decisions would ultimately improve patient outcomes must be investigated in future studies. Larger randomized controlled trials are therefore needed to test the impact of using novel modelling approaches in real-world clinical settings.

## Methods

### Experimental and control groups

We designed a randomized trial focused on CoA to examine the treatment decision impact on practicing cardiologists with experience in treating this condition when they were presented with additional data generated by patient-specific computational modelling.

**Fig. 3:**
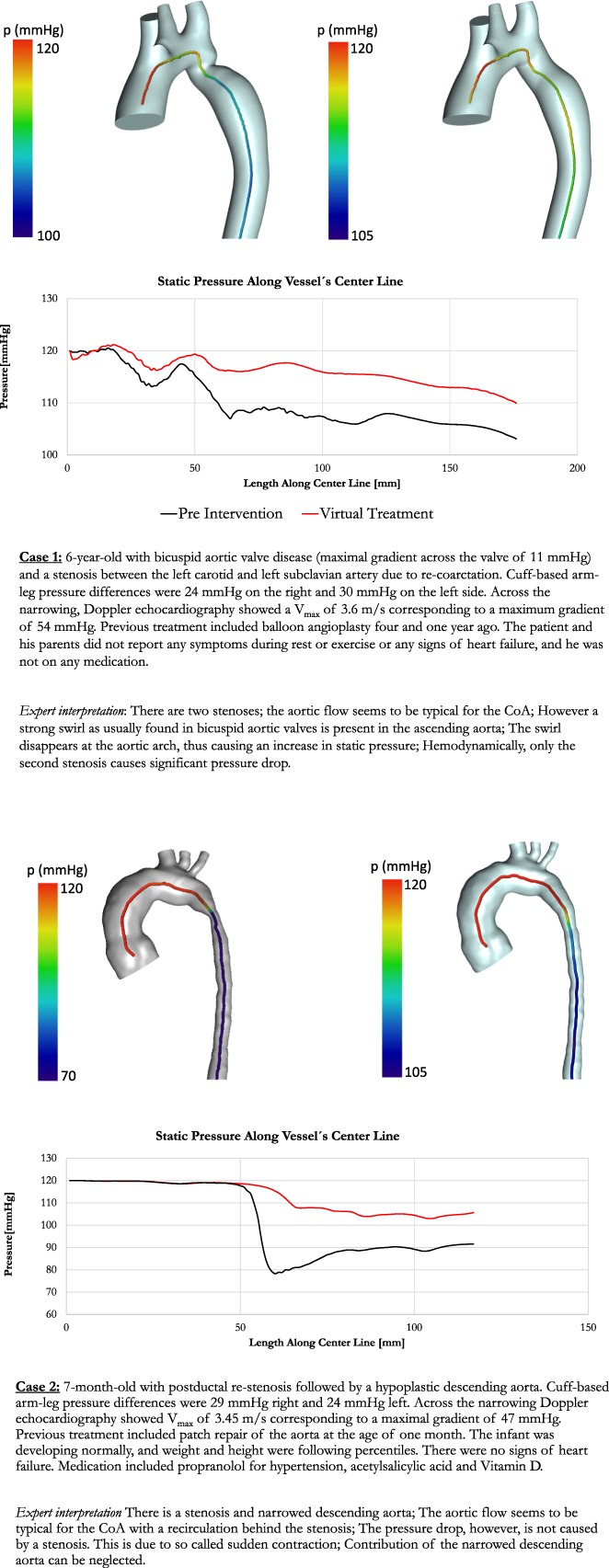

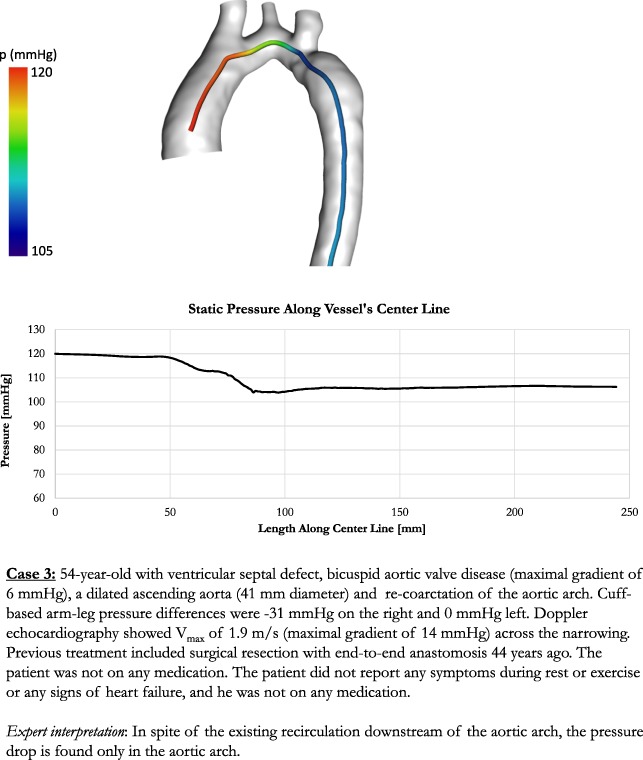
Detailed presentation of cases

We generated two separate datasets for three actual clinical cases. The cases were selected by the clinical project partners in order to reflect clinical heterogeneity, amongst others with respect to age, vessel stenosis (location, degree and length) or presence of associated bicuspid aortic valve. In addition, the selected cases represented differing levels of borderline indication for intervention. Clinical details of the cases are shown in Fig. 3.

We created two datasets for each case. In the first dataset (control), we included conventional parameters (echocardiography and magnetic resonance imaging, MRI) currently recommended as standard diagnostic work-up in clinical practice guidelines (see Table 4). In the second dataset (experimental), in addition to the parameters in the first dataset, we included parameters obtained from clinically validated^7,8^ CFD-based patient-specific modelling. The additional parameters included information about anatomy (geometry, vessel diameter and radius along the centre line) and function (aortic flow field and pressure drop along the centre line). The anatomy of the aorta was segmented using ZIB-Amira (Zuse Institute Berlin, Germany). CFD simulations were based on four-dimensional phase contrast MRI data using Fluent (Version 14.5, ANSYS Inc., Canonsburg, PA, USA). A non-Newtonian blood model was used based on an adapted power law, while turbulence was accounted for by a k-ω SST turbulence model. For spatial discretization, high quality unstructured volume meshes were generated producing approximately one million cells for each patient. Results from flow simulations were validated against catheter measured pressure drop and flow fields measured by four-dimensional phase contrast MRI.

**Table 4:** List of data parameters

**Panel A: Information provided to all participants:**	
• Clinical history	
• Current problems	
• Medication	
• Patient characteristics/physical exam:	
○ Age	
○ Weight	
○ Height	
• Arterial blood pressure	
• Any signs of heart failure	
• Exercise capacity	
**Panel B: Control group**	**Panel C: Experimental group**
*Conventional parameters*	Conventional + patient-specific modeling parameters
Echocardiography:	• All information provided to control group, plus:
• Peak velocity across CoA (m/s)	• Computational fluid dynamics (CFD) modelling information:
• Pressure gradient at CoA (mmHg)	○ Geometry and vessel diameter, including radius along centre line
• Left Ventricle (LV):	○ Screen shots of pre and post interventional virtual stenting modelling outcome
○ End-diastolic diameter (LVEDD in mm)	■ Flow field
• Aortic valve: regurgitation (degree), peak velocity (m/s)	■ Pressure drop along centre line of the ves
MRI:	
• LV:	
○ End-diastolic volume (EDVin mL/m^2^)	
○ End-systolic volume enlarged (ESV in mL/m^2^)	
○ Ejection fraction (EF in %)	
○ Dimensions:	
■ Ascending aorta (mm)	
■ Aortic arch (mm)	
■ Aortic isthmus (mm)	
■ Descending aorta (mm)	

Panel A shows the information provided to all participants. Panel B shows the list of conventional parameters provided to participants in the control group. Panel C shows the list of conventional+patient-specific computational modelling parameters provided to participants in the experimental group

We also presented virtual stenting data for the cases that had borderline indication for intervention. Virtual stenting was performed using a previously introduced interactive tool.^9^ Modelling parameters were presented for the pre-interventional state and a virtual post-interventional state (stenting of the aorta). In addition, applied models were verified to correctly predict pressure drop post-intervention.

**Fig. 4:**
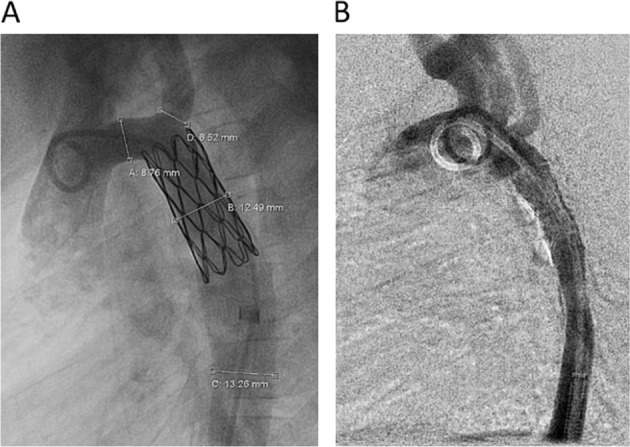
Stent implantation in Cases 1 (**a**) and 2 (**b**)

There was good agreement between modelled and invasive catheterization after stent placement for the two cases for which intervention was indicated according to guidelines (10 vs 12 mmHg and 12 vs. 11 mmHg). The implanted stents did not vary in location, length or diameter compared to the virtual stents used within the simulations shown to the participants (Fig. 4). The remaining case did not receive any intervention, as formal guideline-based treatment indication was not fulfilled. Only modelling information, but no clinical post-treatment images, were presented to the participants for this case.

Clinicians who agreed to participate in the study received information about patient demographic characteristics (age, weight, height), history, symptoms, and clinical status (arterial blood pressure for all four extremities, signs of heart failure, and exercise capacity) for each of the three cases (see Supplementary Information for details). Whether study participants received the first or second dataset was dependent on their group allocation, as described below, as is the recruitment of participants.

### Trial design

The randomized controlled trial design was used to minimise the potential threats to the internal validity of the study. We used a web-based survey platform (Qualtrics) to generate the random allocation sequence (qualtrics.com). Randomized allocation was therefore completed centrally and investigators could not foresee assignment.^32,33^ Given the nature of the intervention, participants were aware of experimental vs. control assignment. However, investigators remained unaware of group allocation until after trial completion.

**Fig. 5:**
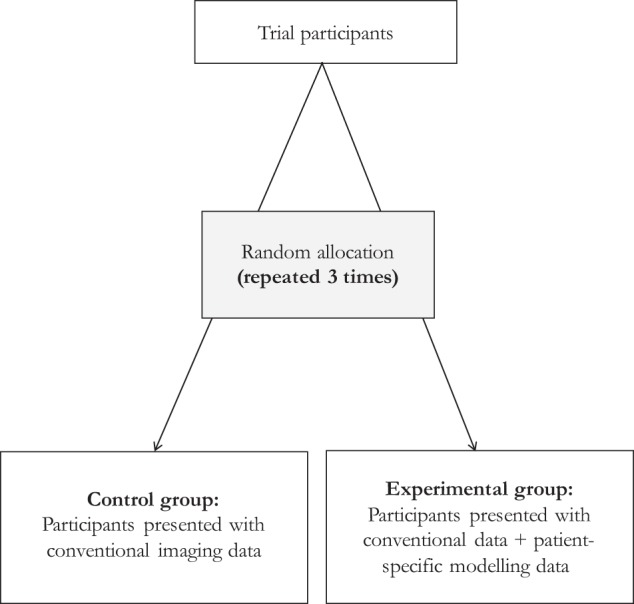
Randomized controlled trial design

Randomization was at the case-level and was therefore repeated three times for each participant (i.e., each participant completed three randomized trials by the end of the study). Thus, it was possible for a trial participant to be randomized to the experimental group for one case and the control group for another. It was also possible for a participant to be repeatedly randomized to the experimental or control group three times. This design ensured that most participants had an opportunity to view CFD simulations at least for some cases. Trial design is shown in Fig. 5.

The target sample size was 118 (59 in each group) on the basis of an anticipated (hypothesized) effect size of 25% difference between the two groups in terms of the primary endpoint. Given the lack of similar evaluations in the literature, our hypothesized effect size was not based on previous empirical studies. We anticipated that an estimated 40% of clinicians presented with the conventional set of parameters (control) as compared to an estimated 65% of clinicians presented with patient-specific modelling parameters (experimental) would decide to recommend either surgery or catheter lab as opposed to follow-up with or without medication, giving an estimated effect size of 25%.

Practicing congenital, pediatric or interventional cardiologists were eligible for inclusion in the study if they had treated patients with coarctation of the aorta during the past 6 months. Clinicians were not eligible to take part in the trial if they were affiliated with the project team running the experiment, (the CARDIOPROOF consortium), or if they had pilot-tested the questionnaire in its previous iterations. Pilot-testing was conducted by cardiologist members of the CARDIOPROOF consortium project and non-consortium members from partner institutions to ensure readability and interpretability of the case summaries and accompanying questions.

We considered participants to have more experience if they had >5 years of experience with decision making in congenital heart disease; >5 coarctation of the aorta cases in the past year; and >5 catheter interventions per year.

Our recruitment strategy had two key elements. First, we manually searched and identified a list of practicing congenital, paediatric and interventional cardiologists in centres with experience in congenital heart disease in Europe and North America. When then contacted them via e-mail. We also obtained access to the member lists of prominent organizations including the Association for European Paediatric and Congenital Cardiology (AEPC). Second, we attended the AEPC Conference and invited eligible clinicians to participate in the experiment using laptops provided by the study team. To incentivize recruitment on site, participants were eligible to voluntarily enter a lottery to win a tablet device, and the CARDIOPROOF consortium made a donation of 10 Euros per participant to a charity organisation (Save the Children). We did not provide any other remuneration to study participants.

### Survey implementation

For the survey, the platform Qualtrics was used to administer the web-based questionnaire.^34^ The first part of the questionnaire presented the clinicians with the study information sheet and asked for their informed consent to take part in the experiment. The second part included a series of questions about participants’ experience level with decision making in congenital heart disease in general, and CoA in particular. Participants indicating that they had no experience with treating patients with CoA were excluded at this stage. Remaining participants were then presented with the three cases, and depending on their group allocation, had access to either the conventional parameters or patient-specific computational modelling parameters. Questions pertaining to study endpoints then followed (see below). At the end of the questionnaire, all participants were asked about their demographic characteristics and geographic region of practice. In total, participants were able to complete the study in approximately 20 min.

Study questions were devised to explore participants’ willingness to recommend intervention in the presented cases depending on the type of information presented to them (experimental vs. control). Participants were first asked for their recommended course of action. Available options were treating the patient with medication and follow-up, leaving the patient untreated and following up, referring the patient to the catheter lab, and referring the patient to surgery. The primary endpoint was “decision to intervene”, referring to a clinician decision to recommend either surgery or catheter lab (collectively, “intervention”) as opposed to follow-up with or without medication (“no intervention”).

### Statistical analysis

We first used descriptive statistics to compare the experimental and control groups at baseline. Differences between study groups in terms of outcomes were then evaluated using Fisher’s exact test for proportions, and chi square analysis with categories adjusted to avoid cells with <5 expected values. Statistical significance was defined as a *P* value of <0.05 assuming a two-tailed hypothesis. All analyzes were repeated for sub-groups according to experience level of participants. Statistical analyzes were performed in STATA (version 14.2; STATA Corp LLC, College Station, TX, USA).

The study was conducted in compliance with the London School of Economics and Political Science Research Ethics Policy and Code of Research Conduct. Due to the hypothetical nature of treatment decisions made as part of this experiment, the study was exempt from ethics review from the London School of Economics and Political Science Research Ethics Committee through the self-certification pathway, and approved by LSE Health. Informed consent was obtained from all participants.

## Supplementary information

Supplementary Information

Supplementary Information

Supplementary Information

## Data Availability

The datasets generated during the current study are available from the corresponding author on reasonable request.

## Supplementary information

**Supplementary information** accompanies the paper on the *npj Digital Medicine* website (10.1038/s41746-019-0085-1).
