# Supplementary material for: Impact of predictive medicine on therapeutic decision making: a randomized controlled trial in congenital heart disease
Source: NPJ Digit Med. 2019 Mar 19;2:17. doi: 10.1038/s41746-019-0085-1 (PMC6550204; doi:10.1038/s41746-019-0085-1)
Supplement: Supplementary file 1 — Supplementary Information [file 41746_2019_85_MOESM1_ESM.pdf]

Information presented to all participants (regardless of group assignment)

CASE 1 of 3

Case presentation

Patient characteristics
Age: 6 years
Gender: male
Weight: 16 kg
Height: 107 cm

Diagnosis
Bicuspid aortic valve
Aortic (re)Coarctation
Stenosis at the aortic arch (between left carotid and left subclavian artery)

History
No corrective surgery
Balloon angioplasty one year ago and four years ago

Current clinical status
Feels good, no clinical signs of heart failure
Good physical excercise capacities

Medication
None

Current diagnostic findings

Arterial pressures (by Riva-Rocci):
Right arm: 119/71 mmHg
Left arm: 122/69 mmHg
Right leg: 95/57 mmHg
Left leg: 92/57 mmHg

Echocardiography:
LV hypertrophy (visually): mild hypertrophy

M-Mode (mm):
LVDd 36, LVDs 2, IVSd 8, IVSs 12, LVPWd 7, LVPWs 9, FS 44%, EF 76%

CW Doppler (distal aortic arch and isthmus) = 3.6 m/s (max gradient = 54 mmHg)
CW Doppler (aortic valve) = 1.7 m/s (max gradient 11 mmHg)

No diastolic run-off

CMR: (see figure 1: MR image of the LV)
LV EDV = 95.2ml/m2
LV ESV = 24.7 ml/m2
LV EF = 74%
Stroke Volume 70.5 ml/m2

Cardiac Index (ml/min) 6.6 l/min/m2

See figure 2 for diameters

Aortic valve: no regurgitation
**Figure 1: MR image of the LV**<br>Fig. 1a: End-diastolic<br>Fig. 1b: End-systolic<br>Fig. 1c: MR angiography
**Figure 2: Geometry and vessel diameters**

CASE 2 of 3

<u>Case presentation</u>

Patient characteristics
Age: 7 months
Gender: male
Weight: 11.9 kg
Height: 81 cm

Diagnosis
Aortic (re)Coarctation

History
Patch repair of coarctation at age of 1 month

Current clinical status
Feels good, no clinical signs of heart failure
Normal development

Medication
Propranolol 3x5mg
ASS 1x15mg
Vitamin D 1x500IU

Current diagnostic findings

<u>Arterial pressures (by Riva-Rocci):</u>
Right arm: 106/57 mmHg
Left arm: 104/56 mmHG
Right leg: 77/45 mmHg
Left leg: 80/39 mmHG

<u>Echocardiography:</u>
LV hypertrophy (visually): mild hypertrophy

M-Mode (mm):
LVIDd 22 mm, LVIDs 15 mm, IVSs 7 mm, PWd 8 mm, PWs 7 mm.
EF 61%, FS 31%

CW Doppler (distal aortic arch and isthmus) = 3.45 m/s (max gradient = 47 mmHg, mean gradient = 18 mmHg)
CW Doppler (aortic valve) = 0.8 m/s

No diastolic run-off

CMR: (see figure 1: MR image of the LV)
LV EDV = 45.3 ml/m2
LV ESV = 11.9 ml/m2
LV EF = 74%
Stroke Volume 33.4 ml/m2

Cardiac Index (ml/min) 3.7 l/min/m2

See figure 2 for diameters

Aortic valve: no regurgitation

Figure 1: MR image of the LV
Fig. 1a: End-diastolic
Fig. 1b: End-systolic

Figure 2: Geometry and vessel diameters

CASE 3 of 3

Case presentation

Patient characteristics
Age: 54 years
Gender: male
Weight: 70 kg
Height: 177 cm

Diagnosis
Bicuspid aortic valve
Aortic (re)Coarctation
VSD (Qp:Qs 1.16;1)
Enlarged ascending aorta (4.1 cm)

History
Surgical resection of Coarctation with end-to-end anastomosis 44 years ago.

Current clinical status
Feels good, no clinical signs of heart failure
Good physical excercise capacities

Medication
None

Current diagnostic findings

Arterial pressures (by Riva-Rocci):
Right arm: 121/81 mmHg
Left arm: 140/79 mmHg
Right leg: 152/71 mmHg
Left leg: 140/76 mmHg

Echocardiography:
LV hypertrophy (visually): mild hypertrophy

M-Mode (mm):<br>LVDd 52, LVDs 2, IVSd 32, IVSs 32, LVPWd 9, LVPWs 13<br>FS 38%, EF 67%<br><br>CW Doppler (distal aortic arch and isthmus) = 1.9 m/s (max gradient = 14 mmHg)<br>CW Doppler (aortic valve) = 1.2 m/s (max gradient 6 mmHg, mean gradient 4 mmHg)<br><br>No diastolic run-off<br><br><u>CMR:</u><br>See figure 1 for MR image of the LV<br><br>See figure 2 for diameters<br><br>Aortic valve: regurgitation 2.3%
**Figure 1: MR image of the LV**<br>Fig. 1a: End-diastolic<br>Fig. 1b: End-systolic
**Figure 2: Geometry and vessel diameters**
